# Risk of Postoperative Gastrointestinal Bleeding and Its Associated Factors: A Nationwide Population-Based Study in Korea

**DOI:** 10.3390/jpm11111222

**Published:** 2021-11-18

**Authors:** Sang Hyuck Kim, Kyungdo Han, Gunseog Kang, Seung Woo Lee, Chi-Min Park, Jongho Cho, Jung Won Choi, Se Jun Park, Minyong Kang, Tae Jun Kim, Seo-Hee Hong, Yong-Chol Kwon, Junhee Park, Dongwook Shin

**Affiliations:** 1 Department of Family Medicine, Bumin Hospital, Seoul 07590, Korea; fmmdshk@naver.com; 2 Department of Statistics and Actuarial Science, Soongsil University, Seoul 06978, Korea; hkd917@naver.com (K.H.); gskang@ssu.ac.kr (G.K.); 3 Department of Biostatistics, College of Medicine, Catholic University, Seoul 06591, Korea; ghdk32@naver.com; 4 Department of Surgery, Samsung Medical Center, Sungkyunkwan University School of Medicine, Seoul 06351, Korea; dr99.park@samsung.com; 5 Department of Cardiovascular and Thoracic Surgery, Samsung Medical Center, Sungkyunkwan University School of Medicine, Seoul 06351, Korea; Jongho.cho@gmail.com; 6 Department of Neurosurgery, Samsung Medical Center, Sungkyunkwan University School of Medicine, Seoul 06351, Korea; jungwon8090.choi@samsung.com; 7 Department of Orthopedic Surgery, Samsung Medical Center, Sungkyunkwan University School of Medicine, Seoul 06351, Korea; sejunos.park@samsung.com; 8 Department of Urology, Samsung Medical Center, Sungkyunkwan University School of Medicine, Seoul 06351, Korea; m79.kang@samsung.com; 9 Department of Gastroenterology, Samsung Medical Center, Sungkyunkwan University School of Medicine, Seoul 06351, Korea; tj23.kim@samsung.com; 10 Medical Affairs, Pfizer Essential Health Business Unit, Pfizer Pharmaceuticals Korea Limited, Seoul 04631, Korea; Seo-Hee.Hong@viatris.com (S.-H.H.); Yong-Chol.Kwon@viatris.com (Y.-C.K.); 11 Department of Family Medicine/Supportive Care Center, Samsung Medical Center, Seoul 06351, Korea; junhee.park26@gmail.com; 12 Department of Clinical Research Design & Evaluation, Samsung Advanced Institute for Health Science & Technology (SAIHST), Sungkyunkwan University, Seoul 06355, Korea

**Keywords:** postoperative bleeding, bleeding risk, analgesics, pain control

## Abstract

Postoperative gastrointestinal bleeding (PGIB) is a serious complication with expensive medical costs and a high mortality rate. This study aims to analyze the incidence of PGIB and its associated factors, including its relationship with postoperative analgesic use. Patients aged ≥20 years who received various kinds of surgery from 2013 to 2017 were included (*n* = 1,319,807). PGIB was defined by admission with ICD-10 codes of gastrointestinal bleeding plus transfusion within 2 months after surgery. A total of 3505 (0.27%) subjects had PGIB, and the incidence was much higher for those who underwent major gastrointestinal and major cardiovascular surgery (1.9% for both), followed by major head and neck (0.7%), major genitourinary (0.5%), and orthopedic surgery (0.45%). On multivariate analysis, older age, male sex, lower income, comorbidities, peptic ulcer disease, and congestive heart failure were associated with a higher risk of gastrointestinal bleeding. Among analgesics, steroid use was associated with increased postoperative bleeding risk (adjusted OR: 1.36, 95% CI: 1.25–1.48). Acetaminophen/nonsteroidal anti-inflammatory drugs, cyclooxygenase 2 inhibitors, anticonvulsants, antidepressants, and opioids were not associated with increased risk. PGIB is considerable for major surgeries, and its risk should be considered, especially for patients with older age and comorbidities and use of steroids.

## 1. Introduction

Postoperative gastrointestinal bleeding (PGIB) is a critical complication associated with high morbidity and mortality. In a study performed in two tertiary hospitals in the US from 1982 to 1994, the estimated mortality of PGIB after major surgery was as high as 31% [1], and its incidence was estimated to be 0.39% [1]. However, the incidence of PGIB varies widely according to the characteristics of the study population and the definition of PGIB. A review by Hiramoto et al. estimated the incidence of clinically important PGIB, defined as needing transfusion or with unstable vital signs, to be 0.6–6% [2]. However, there has been a paucity of research on the incidence of PGIB across various clinical settings, especially for the type and extent of surgery and various patients’ characteristics. Moreover, some analgesics, such as nonsteroidal anti-inflammatory drugs (NSAIDs), are allegedly associated with gastrointestinal bleeding [3]. Postoperative pain has impacts on patient satisfaction, quality of life, daily activities, and personal/social costs [3,4,5], and thus, adequate postoperative pain control is essential for reducing in-hospital stay, respiratory/cardiovascular morbidity, and even mortality [6]. Various mechanisms are involved in postoperative pain, and thus, the approach to this is multimodal, involving several different analgesics such as NSAIDs, cyclooxygenase 2 inhibitors (COX-2i), anticonvulsants, antidepressants, opioids, and steroids [7,8]. To optimize the use of postoperative analgesics, it is important to consider the benefits and risks of each, especially in terms of PGIB risk.

This study used nationwide, population-based data in South Korea to analyze the incidence of PGIB and its associated factors, including its relationship with postoperative analgesic use.

## 2. Materials and Methods

### 2.1. Data Source

We used claims data from the National Health Insurance Service (NHIS). In South Korea, a nationwide single-insurer system was launched in 2000, and the National Health Insurance covers approximately 98% of the overall population [9,10]. After passing the institutional approval procedure, the NHIS provides a customized dataset from the original claims data of both inpatients and outpatients. A detailed explanation of the data structure and characteristics is available elsewhere [10,11]. This study was approved by the institutional review board of Sungkyunkwan University (IRB No. SKKU 2019-12-004) and Bumin Hospital (IRB No. 202002-CTDG-002).

### 2.2. Study Population

Patients aged ≥20 years who received various kinds of surgery from 1 January 2013 to 31 December 2017, were included. A total of 1,319,807 subjects were included for the analysis. Surgery types were categorized as follows: (1) head and neck, major (e.g., brain tumor surgery); (2) head and neck, minor (e.g., sinus surgery); (3) cardiovascular, major (e.g., coronary artery bypass or valve surgery); (4) gastrointestinal, major (e.g., gastrectomy, hepatectomy, and colectomy); (5) gastrointestinal, minor (e.g., appendectomy and cholecystectomy); (6) genitourinary, major (e.g., prostatectomy and nephrectomy); (7) gynecological, major (e.g., hysterectomy); (8) gynecological, minor (e.g., cesarean section); (9) orthopedic, major (e.g., hip replacement); (10) orthopedic, minor (e.g., endoscopic spinal surgery); and (11) others (all minor, e.g., hemorrhoidectomy and thyroidectomy). If more than one surgery was performed, only the first surgery was considered for the analysis. Detailed surgery profiles of each category are provided as supplementary material (Supplementary Table S1).

### 2.3. Definition of Variables

#### 2.3.1. Postoperative Gastrointestinal Bleeding

We defined PGIB events based on the relevant ICD-10 codes upon admission (“K226,” “K250”, “K252”, “K254”, “K256”, “K260”, “K262”, “K264”, “K266”, “K270”, “K272”, “K274”, “K276”, “K280”, “K282”, “K284”, “K286”, “K290”, “K625”, “K920”, “K921”, and “K922”). In consideration of a previous study, we further defined PGIB as having the aforementioned ICD-10 codes plus a procedure code for blood transfusion, which signifies clinically important bleeding [2]. Only gastrointestinal bleeding events occurring within 2 months after surgery were included in the analysis because it has previously been reported that the interval between operation and PGIB was between 1 and 55 days [1]. Moreover, cases of PGIB occurring during the same admission period for the surgery itself were considered in-hospital bleeding.

#### 2.3.2. Preoperative Comorbidities and Antiplatelet/Anticoagulant Use

Comorbidities were defined by ICD 10 code and prescription information administered during a certain period before surgery, considering the chronological nature of a disease. Hypertension (“I10”, “I11”, “I12”, “I13”, and “I15”), diabetes mellitus (“E11”, “E12”, “E13”, and “E14”), and dyslipidemia (“E78”) were defined by their diagnosis codes plus the presence of relevant drug prescription for each comorbidity (i.e., antihypertensive, antidiabetic, and lipid lowering medications, respectively) within 1 year before surgery. Chronic liver disease (“K72”, “K73”, and “K74”), chronic kidney disease (“N18” and “N19”), and congestive heart failure (“I50”) were defined according to diagnosis code within 1 year before surgery. Peptic ulcer disease (“K25”, “K26”, “K27”, and “K28”) and gastrointestinal reflux disease (“K21”) were defined according to diagnosis code within 3 months before surgery.

Prescription of antiplatelet and anticoagulant medications was considered in the analyses as they are well-known risk factors for gastrointestinal bleeding [12,13]. Those who were prescribed antiplatelet or anticoagulant medications within 1 year before surgery were considered to be on that medication at the time of surgery. The antiplatelet drugs included aspirin, clopidogrel, prasugrel, ticagrelor, limaprost, triflusal, dipyridamole, beraprost, cilostazol, sarpogrelate, ticlopidine, and *Ginko* *biloba* leaf extract. The anticoagulants included warfarin, rivaroxaban, dabigatran, apixaban, and edoxaban.

#### 2.3.3. Postoperative Medication Use

Postoperative analgesics were defined as prescription of each analgesic drug category at least once within 2 weeks after surgery. Analgesics were categorized as follows: (1) acetaminophen and NSAID (e.g., naproxen and ibuprofen), (2) COX-2i (e.g., celecoxib and etoricoxib), (3) anticonvulsant (e.g., carbamazepine, gabapentin, and pregabalin), (4) antidepressant (e.g., amitriptyline and duloxetine), (5) opioid (e.g., tramadol, morphine, oxycodone, hydrocodone, and tapentadol), and (6) steroid (oral or injection; e.g., dexamethasone and methylprednisolone). Because the NHIS gave the information regarding acetaminophen and NSAIDs indistinguishably combined as one (there was an internal policy not to open the data of a single drug, acetaminophen, in this case), we had no choice but to group the two analgesics.

Postoperative gastrointestinal drug use was also considered because this can be protective for PGIB. This was defined as the prescription of each gastrointestinal drug category at least once within 2 weeks after surgery. On the basis of the drug classification of the Korean Food and Drug Administration, drugs were categorized as follows: (1) mucosal protective agent (e.g., sodium alginate, rebamipide, sucralfate, teprenone, bismuth, troxipide, ecabet sodium, irsogladine maleate, polaprezinc, and *Artemisia* *argyi* folium extracts), (2) H2 receptor antagonist (e.g., cimetidine, ranitidine, famotidine, and lafutidine), (3) proton pump inhibitor (PPI; e.g., omeprazole, esomeprazole, rabeprazole, and lansoprazole), and (4) antacid (almagate, calcium carbonate, diomagnite, magnesium aluminum, magnesium hydroxide, and magnesium oxide).

### 2.4. Statistical Analysis

Univariate analysis was performed for each of the baseline characteristics according to PGIB event, specifically using *t*-test for continuous variables and chi-square test for categorical variables. The total PGIB and in-hospital PGIB incidence, as well as the hospitalization period with/without PGIB according to the types of surgery, were calculated. To compare the means of hospitalization period with/without PGIB *t*-test was conducted. To explore the factors associated with PGIB, we performed three serial logistic regression analyses: (1) Model 1 is a nonadjusted analysis for each variable, (2) Model 2 includes baseline characteristics before surgery (i.e., age, sex, income, residence, comorbid conditions, and antiplatelet/anticoagulant drug use) and surgery type, and (3) Model 3 additionally included postoperative drug use, including analgesic use and gastrointestinal medication.

In South Korea, the reimbursement criteria for COX-2i were very restrictive and were mainly used for patients aged 60 years or older until 1 December 2017. Therefore, elderly subjects who had increased postoperative bleeding risk were usual candidates for COX-2i. In fact, COX-2i was mostly used in those aged 60 years or older (*n* = 42,992, 83.14%; data not shown). Therefore, we also performed a subgroup analysis for subjects aged 65 years or older to reduce potential indication bias by age.

## 3. Results

### 3.1. Baseline Characteristics

Among the overall study population, 3505 (0.27%) subjects had PGIB. The mean age of subjects with bleeding was higher than that of subjects without bleeding (67.36 vs. 49.87 years, respectively). Postoperative bleeding was more frequent as age increased, with 72.18% of bleeding cases occurring in those aged 60 years and older. PGIB was more frequently observed in males than in females (0.36% vs. 0.20%, respectively) and in low-income patients than in high-income ones (0.37% vs. 0.24%, respectively). Antiplatelet/anticoagulant users, subjects with comorbid conditions, and those given postoperative drugs for pain/peptic ulcer all had more frequent PGIB, except for acetaminophen/NSAID users (all *p* < 0.001) (Table 1).

### 3.2. Surgery Type and Postoperative Gastrointestinal Bleeding

Major surgeries had relatively more frequent PGIB. Specifically, major cardiovascular surgery (1.89%) and major gastrointestinal surgery (1.92%) had the most frequent PGIB, followed by major head and neck (0.71%), major genitourinary (0.54%), and orthopedic (0.45%) surgeries.

Most postoperative bleeding occurred during the hospitalization period for the surgery itself (75.44%; 2644 out 3505 PGIB cases). The overall hospitalization period was significantly prolonged if gastrointestinal bleeding occurred (22.94 vs. 7.59 days, *p* < 0.001). Among subjects who experienced PGIB, the hospitalization period was generally more than 20 days for most major surgeries and even over 30 days for major head and neck surgeries (Table 2).

### 3.3. Risk Factors for Postoperative Gastrointestinal Bleeding

The risk of PGIB was higher as age increased. A lower risk of PGIB was observed in females than in males (adjusted odds ratio (aOR): 0.72, 95% confidence interval (CI): 0.67–0.77) and in people with a lower income than in those with a higher income (aOR: 1.25, 95% CI: 1.16–1.35).

The following comorbidities were associated with increased PGIB risk: diabetes mellitus (aOR: 1.34, 95% CI: 1.24–1.45), hypertension (aOR: 1.31, 95% CI: 1.20–1.42), chronic liver disease (aOR: 1.54, 95% CI: 1.38–1.73), congestive heart failure (aOR: 1.78, 95% CI: 1.60–1.98), and peptic ulcer disease (aOR: 1.20, 95% CI: 1.11–1.30). On the other hand, gastrointestinal reflux (aOR: 0.82, 95% CI: 0.76–0.89) and dyslipidemia (aOR: 0.86, 95% CI: 0.79–0.93) were inversely associated with PGIB risk. Preoperative anticoagulant use was associated with increased risk (aOR: 1.20, 95% CI: 1.07–1.35), but antiplatelet use was not (aOR: 1.02, 95% CI: 0.94–1.11).

When other minor surgeries were used as a reference, the following major surgery types were associated with significant increased bleeding risk: head and neck (aOR: 3.73, 95% CI: 2.73–5.10), cardiovascular (aOR: 6.05, 95% CI: 5.02–7.29), gastrointestinal (aOR: 10.96, 95% CI: 9.51–12.64), and orthopedic (aOR: 2.13, 95% CI: 1.85–2.44). By contrast, major gynecological surgery (aOR: 0.69, 95% CI: 0.45–1.07) was not associated with increased bleeding risk.

Postoperative steroid use, compared to nonuse, was associated with a higher risk of PGIB (aOR: 1.29, 95% CI: 1.20–1.39). However, acetaminophen/NSAID use (aOR: 0.75, 95% CI: 0.70–0.81) and opioid use (aOR: 0.89, 95% CI: 0.82–0.97) both showed lower PGIB risk. Use of other pain control drugs, such as COX-2is (aOR: 1.08, 95% CI: 0.95–1.22), anticonvulsants (aOR: 9.93, 95% CI: 0.80–1.09), and antidepressants (aOR: 0.94, 95% CI: 0.76–1.16), was not associated with PGIB risk (Table 3).

Subjects who took the following gastrointestinal drugs for peptic ulcer showed a significantly increased risk of PGIB: mucosal protective agents (aOR: 1.89, 95% CI: 1.76–2.02), H2 receptor antagonists (aOR: 1.17, 95% CI: 1.09–1.26), proton pump inhibitors (aOR: 3.00, 95% CI: 2.78–3.23), and antacids (aOR: 1.29, 95% CI: 1.12–1.39) (Table 3).

The results of the subgroup analysis for patients aged 65 years and older were consistent with those of the main analysis (Table 4).

## 4. Discussion

To the best of our knowledge, this is the first study to report the incidence of PGIB-associated factors using nationwide, population-based data across various surgery types. The incidence was higher for major surgeries, such as gastrointestinal and cardiovascular surgery. PGIB was substantially frequent in elderly subjects, and PGIB significantly prolonged the hospitalization period.

Our study showed a 0.27% risk of PGIB for overall surgery, but this was much higher for major surgeries (as high as 2% for major cardiovascular and gastrointestinal surgery). Although there is little research to compare our results, it is generally consistent with the results of previous studies that reported PGIB in 0.39% of major surgeries in a US university hospital [1] and in 1.01% of cardiovascular surgeries in Canada [14]. Similar to previous studies, the risk of PGIB was increased in the elderly and those with comorbidities [15]. Around 3/4 of PGIB cases occurred during the hospitalization period for the surgery, and this significantly lengthened hospital stay. This suggests the clinical and economic importance of PGIB because prolonging hospital stay would increase medical costs and chances for other complications and comorbidities [3,5,6].

In our study, age was significantly associated with PGIB, and patients aged ≥70 years showed around 20 times the risk of those who were in their 20s. Because both age and the high prevalence of various comorbidities among the elderly are associated with PGIB, morbidity and mortality with gastrointestinal bleeding are substantial [12,13,16]. Because PGIB has a high mortality rate and increases the length of hospital stay, efforts to reduce this risk are warranted during this critical period, especially among the elderly [1].

Diabetes mellitus, hypertension, chronic liver disease, chronic kidney disease, and congestive heart failure were all associated with increased PGIB risk. Those comorbidities are associated with concomitant multidrug use, disruption of the coagulation cascade, alteration of gastrointestinal function, and variceal and peptic ulcer bleeding [17,18,19,20,21,22]. Generally, antiplatelet and anticoagulants are known to increase gastrointestinal bleeding [19,23,24]. In our study, only anticoagulants were associated with increased risk; antiplatelet drugs were not. According to the guidelines for perioperative antiplatelet/anticoagulant use, anticoagulants are more likely to have dose reductions or changes in formulation rather than being discontinued during the perioperative period, considering thromboembolic events. On the other hand, antiplatelet use for primary prevention might have been discontinued perioperatively. Furthermore, several cases of antiplatelet use for secondary prevention might have been discontinued considering the risk of bleeding [25,26]. These clinical practice patterns might have affected the results of the present study. Furthermore, although some patients discontinued antiplatelet medication several months before surgery, we considered them to have been on such medication based on our operational definition, which might have produced misclassification bias. Thus, we need to be more careful when caring for subjects with these risk factors.

Clinically speaking, the most interesting topic of our study is whether postoperative analgesic use increases the risk of PGIB. In particular, COX-2i is well known for its less frequent gastrointestinal side effects, and it was expected to be associated with a lower risk of PGIB [27,28]. However, in the present study, the expected effect of COX-2i to reduce the risk was not evident. On the other hand, acetaminophen/NSAID users had a significantly lower risk of gastrointestinal bleeding. Because NSAIDs are associated with gastropathy and gastrointestinal bleeding in the postoperative period [1,3], it is likely that the increased risk by NSAID was offset by the decreased risk of acetaminophen user. For the same reason, we could not directly compare the effect of NSAIDs and COX-2is on PGIB. We also suspected that the different association of these two drug classes on PGIB was a result of indication bias, which means that COX-2i prescriptions were focused on high-risk groups, but the consistent results from stratified analysis of patients aged ≥65 years indicated otherwise. Nevertheless, it can be concluded that COX-2i does not seem to increase the PGIB risk.

Opioid use was associated with slightly lower bleeding risk. Opioids are indicated for moderate to severe pain, especially for cancer patients. The reason for this lower risk was not clear, and further studies are needed to explain this. Anticonvulsants and antidepressants, which are commonly used as adjuvant analgesics to opioids, were not significantly associated with PGIB. To our knowledge, these agents were not reported to increase PGIB risk, consistent with our results. On the other hand, steroid use was associated with increased PGIB risk, as expected [29]. Steroids are sometimes used as adjuvant analgesics to decrease opioid use [3,29] and are used for brain edema and increased intracranial pressure [30]. Because steroids are well-known ulcerogenic agents that increase gastrointestinal perforation and bleeding, caution should be taken during steroid use.

Unexpectedly, postoperative gastrointestinal drug use was associated with increased PGIB. These drugs may be prescribed prophylactically for high-risk patients with symptoms such as dyspepsia and gastropathy, including peptic ulcer and gastritis. These gastrointestinal drugs might have been started after PGIB occurred rather than before. Such reverse causality from our retrospective study design would explain this unexpected result.

There are some limitations in this study. First, the definition of PGIB was operatively made with claims data. Because gastrointestinal bleeding is common in acute stress events, the prevalence would be much higher if a different definition (e.g., not requiring transfusion) was applied. However, because our definition was based on previous literature, it can be considered clinically meaningful. Second, the analyses on the effects of pre- and postoperative drug use were not very accurate. Because there were too many situations to consider, we only considered whether the drug was prescribed at a certain timing before or after the surgery. Specifically, drug dose and duration of administration were not fully taken into account. Third, in the case of PGIB that occurred during inpatient care, claims for the reimbursement of transfusion might not be made on the same day of the event but might be made several days later during the admission episode. Therefore, the exact day of PGIB might be different from the day of our PGIB definition. Therefore, it could be ambiguous whether analgesic or gastrointestinal drug administration was made before or after the PGIB event. Fourth, while we defined our outcome as clinically important GI bleeding, this does not necessarily mean severe bleeding. A better definition with more detailed clinical data would enrich the analyses but was not possible here due to data constraints. Finally, as mentioned in the Methods section, we could not obtain separate drug data for acetaminophen and NSAIDs due to the data provision policy of the NHIS. Despite these limitations, a large and representative sample enabled us to produce reliable estimates on the prevalence of clinically meaningful PGIB. Given the paucity of research in this area, our study suggests the presence of many risk and protective factors for PGIB, which can stimulate further research on this topic.

## 5. Conclusions

We showed that there is considerable risk of PGIB, especially in major surgeries, and this risk should be considered, especially for patients who are older and have comorbidities. Postoperative analgesic use was generally not associated with an increased risk of postoperative bleeding, except for steroid use, ensuring that postoperative pain can be adequately controlled without an increased risk of PGIB.

## Figures and Tables

**Table 1:** Baseline characteristics of the study population (*n*, %) *.

	Total		Postoperative Gastrointestinal Bleeding			
			No		Yes	
	*n* = 1,319,807		*n* = 1,316,302		*n* = 3505	
Age (years)						
Mean ± SD	49.92 ± 17.18		49.87 ± 17.16		67.36 ± 15.06	
20–29	160,689	(12.18)	160,616	(99.95)	73	(0.05)
30–39	278,136	(21.07)	278,004	(99.95)	132	(0.05)
40–49	238,445	(18.07)	238,179	(99.89)	266	(0.11)
50–59	236,871	(17.95)	236,367	(99.79)	504	(0.21)
60–69	188,721	(14.30)	188,065	(99.65)	656	(0.35)
≥70	216,945	(16.44)	215,071	(99.14)	1874	(0.86)
Sex						
Male	523,657	(39.68)	521,762	(99.64)	1895	(0.36)
Female	796,150	(60.32)	794,540	(99.80)	1610	(0.20)
Income status						
Upper 80%	1,081,113	(81.91)	1,078,490	(99.76)	2623	(0.24)
Lower 20%	238,694	(18.09)	237,812	(99.63)	882	(0.37)
Residence						
Urban	596,306	(45.18)	594,827	(99.75)	1479	(0.25)
Rural	723,501	(54.82)	721,475	(99.72)	2026	(0.28)
Preoperative comorbidities						
Diabetes Mellitus	128,576	(9.74)	127,499	(99.16)	1077	(0.84)
Hypertension	350,131	(26.53)	347,919	(99.37)	2212	(0.63)
Dyslipidemia	235,077	(17.81)	233,816	(99.46)	1261	(0.54)
Chronic Liver Disease	40,368	(3.06)	40,011	(99.12)	357	(0.88)
Chronic Kidney Disease	19,454	(1.47)	19,056	(97.95)	398	(2.05)
Congestive Heart Failure	31,948	(2.42)	31,449	(98.44)	499	(1.56)
Peptic Ulcer Disease	159,385	(12.08)	158,373	(99.37)	1012	(0.63)
Gastroesophageal Reflux	260,445	(19.73)	259,259	(99.54)	1186	(0.46)
Preoperative drug use						
Antiplatelet User	186,437	(14.13)	185,282	(99.38)	1155	(0.62)
Anticoagulant User	42,744	(3.24)	42,341	(99.06)	403	(0.94)
Postoperative analgesic use						
Acetaminophen/NSAIDs	759,784	(57.57)	758,047	(99.77)	1737	(0.23)
Cyclooxygenase 2 inhibitor	51,709	(3.92)	51,370	(99.34)	339	(0.66)
Anticonvulsant	37,161	(2.82)	36,971	(99.49)	190	(0.51)
Antidepressant	18,847	(1.43)	18,753	(99.50)	94	(0.50)
Opioid	425,756	(32.26)	423,488	(99.47)	2268	(0.53)
Steroid	188,512	(14.28)	187,633	(99.53)	879	(0.47)
Postoperative gastrointestinal medication use						
Mucosal Protective Agents	324,242	(24.57)	322,659	(99.51)	1583	(0.49)
H2 Receptor Antagonist	416,323	(31.54)	414,982	(99.68)	1341	(0.32)
Proton Pump Inhibitor	105,806	(8.02)	104,538	(98.80)	1268	(1.20)
Antacid	364,288	(27.60)	362,989	(99.64)	1299	(0.36)

* *p* values for each characteristic are below 0.001 (*t*-test for continuous variables and chi-square test for categorical variables).

**Table 2:** Gastrointestinal bleeding prevalence and hospitalization period by surgery type.

Surgery Type	Total	Total Bleeding (n, %)		In-Hospital Bleeding (n, %)		Hospitalization Period (Days) with Bleeding (Mean ± SD)		Hospitalization Period (Days) without Bleeding (Mean ± SD)	
Head and Neck (major)	7007	50	(0.71)	46	(0.66)	34.59	±25.67	16.52	±12.08
Head and Neck (minor)	164,792	48	(0.03)	11	(0.01)	17.73	±17.16	2.06	±2.43
Cardiovascular (major)	15,379	290	(1.89)	232	(1.51)	23.26	±10.35	16.37	±8.98
Gastrointestinal (major)	60,213	1156	(1.92)	1020	(1.69)	21.98	±14.48	13.73	±8.35
Gastrointestinal (minor)	160,621	287	(0.18)	187	(0.12)	19.21	±15.76	6.16	±4.26
Genitourinary (major)	13,162	71	(0.54)	57	(0.43)	24.40	±13.97	12.54	±7.69
Gynecological (major)	50,797	22	(0.04)	12	(0.02)	26.92	±15.23	7.24	±3.94
Cesarean Section (minor)	162,544	9	(0.01)	7	(0.00)	8.43	±12.59	6.50	±1.47
Orthopedic (major)	276,446	1245	(0.45)	953	(0.34)	25.44	±15.81	16.16	±10.37
Orthopedic (minor)	2473	2	(0.08)	2	(0.08)	21.00	±1.41	6.15	±5.88
Others (minor)	406,373	325	(0.08)	117	(0.03)	12.04	±11.56	3.55	±3.05
Total	1,319,807	3505	(0.27)	2644	(0.20)	22.94	±15.28	7.59	±7.91

All *p* values for hospitalization period between subjects with and without bleeding are below 0.001.

**Table 3:** Logistic regression analysis for postoperative gastrointestinal bleeding risk (odds ratio, 95% confidence interval).

	Model 1		Model 2		Model 3	
Age, years						
20–29	Reference		Reference		Reference	
30–39	1.045	(0.785, 1.391)	1.109	(0.832, 1.478)	1.076	(0.807, 1.435)
40–49	2.457	(1.896, 3.184)	1.268	(0.975, 1.649)	1.201	(0.923, 1.563)
50–59	4.691	(3.670, 5.997)	1.436	(1.115, 1.851)	1.346	(1.045, 1.735)
60–69	7.675	(6.026, 9.775)	1.565	(1.213, 2.019)	1.464	(1.135, 1.889)
≥70	19.171	(15.173, 24.224)	3.276	(2.549, 4.210)	3.011	(2.343, 3.869)
Sex						
Male	Reference		Reference		Reference	
Female	0.558	(0.522, 0.596)	0.740	(0.689, 0.794)	0.716	(0.667, 0.769)
Income						
<20%	1.525	(1.413, 1.646)	1.289	(1.193, 1.392)	1.253	(1.159, 1.354)
≥20%	Reference		Reference		Reference	
Residence						
Urban	Reference		Reference		Reference	
Rural	1.129	(1.056, 1.208)	0.997	(0.932, 1.067)	0.990	(0.925, 1.060)
Comorbidity *						
Diabetes Mellitus	4.137	(3.850, 4.446)	1.384	(1.278, 1.497)	1.341	(1.238, 1.452)
Hypertension	4.762	(4.445, 5.100)	1.330	(1.225, 1.444)	1.306	(1.203, 1.418)
Dyslipidemia	2.602	(2.428, 2.788)	0.869	(0.804, 0.940)	0.858	(0.794, 0.928)
Chronic Liver Disease	3.617	(3.241, 4.038)	1.629	(1.456, 1.823)	1.541	(1.376, 1.726)
Chronic Kidney Disease	8.721	(7.849, 9.690)	2.916	(2.601, 3.269)	2.655	(2.362, 2.985)
Peptic Ulcer Disease	2.968	(2.758, 3.194)	1.246	(1.153, 1.347)	1.199	(1.109, 1.296)
Congestive Heart Failure	6.784	(6.167, 7.463)	1.884	(1.694, 2.094)	1.779	(1.599, 1.979)
Gastroesophageal Reflux	2.085	(1.944, 2.237)	0.952	(0.885, 1.025)	0.822	(0.763, 0.886)
Antiplatelet/Anticoagulant *						
Antiplatelet User	3.000	(2.796, 3.220)	1.059	(0.978, 1.146)	1.023	(0.944, 1.108)
Anticoagulant User	3.909	(3.522, 4.338)	1.288	(1.149, 1.443)	1.204	(1.073, 1.352)
By surgery types						
Head and Neck (major)	8.979	(6.661, 12.105)	6.499	(4.814, 8.774)	3.734	(2.732, 5.104)
Head and Neck (minor)	0.364	(0.269, 0.493)	0.418	(0.309, 0.566)	0.352	(0.259, 0.478)
Cardiovascular (major)	24.012	(20.479, 28.155)	9.104	(7.659, 10.821)	6.051	(5.024, 7.289)
Gastrointestinal (major)	24.456	(21.618, 27.667)	11.891	(10.434, 13.551)	10.964	(9.513, 12.636)
Gastrointestinal (minor)	2.236	(1.908, 2.621)	1.826	(1.556, 2.143)	1.652	(1.403, 1.945)
Genitourinary (major)	6.776	(5.239, 8.765)	2.424	(1.862, 3.156)	1.599	(1.215, 2.104)
Gynecological (major)	0.541	(0.351, 0.834)	0.602	(0.390, 0.928)	0.693	(0.448, 1.070)
Cesarean Section (minor)	0.069	(0.036, 0.134)	0.166	(0.085, 0.324)	0.198	(0.101, 0.387)
Orthopedic (major)	5.652	(5.002, 6.387)	2.742	(2.403, 3.128)	2.127	(1.852, 2.442)
Orthopedic (minor)	1.011	(0.252, 4.063)	0.983	(0.244, 3.950)	0.839	(0.208, 3.376)
Others (minor)	Reference		Reference		Reference	
Postoperative drugs user *						
Acetaminophen/NSAIDs	0.723	(0.677, 0.773)			0.749	(0.696, 0.807)
Cyclooxygenase 2 inhibitor	2.637	(2.357, 2.950)			1.077	(0.949, 1.223)
Anticonvulsant	1.984	(1.714, 2.298)			0.931	(0.799, 1.085)
Antidepressant	1.908	(1.553, 2.342)			0.937	(0.759, 1.156)
Opioid	3.865	(3.606, 4.143)			0.892	(0.824, 0.965)
Steroid	2.014	(1.865, 2.174)			1.361	(1.250, 1.482)
Mucosal Protective Agents	2.537	(2.374, 2.712)			1.886	(1.760, 2.021)
H2 Receptor Antagonist	1.346	(1.257, 1.441)			1.171	(1.090, 1.259)
Proton Pump Inhibitor	6.571	(6.132, 7.042)			3.000	(2.784, 3.233)
Antacid	1.546	(1.444, 1.656)			1.292	(1.199, 1.392)

Model 1 is a nonadjusted analysis for each variable, Model 2 includes baseline characteristics before surgery (i.e., age, sex, income, residence, comorbid conditions, and antiplatelet/anticoagulant drug use) and surgery type, and Model 3 additionally included postoperative drug use, including analgesic use and gastrointestinal medication. * Nondrug users and without comorbidities are references.

**Table 4:** Logistic regression analysis for subjects aged 65 or more (odds ratio, 95% confidence interval).

	Model 1		Model 2		Model 3	
Sex						
Male	Reference		Reference		Reference	
Female	0.746	(0.686, 0.811)	0.831	(0.758, 0.910)	0.813	(0.741, 0.891)
Income low (20%)						
<20%	1.303	(1.184, 1.434)	1.278	(1.160, 1.408)	1.242	(1.126, 1.369)
≥20%	Reference		Reference		Reference	
Residence						
Urban	Reference		Reference		Reference	
Rural	0.945	(0.868,1.028)	0.912	(0.837, 0.993)	0.911	(0.836, 0.993)
Comorbidity *						
Diabetes Mellitus	1.727	(1.582, 1.886)	1.306	(1.19, 1.434)	1.278	(1.164, 1.404)
Hypertension	1.748	(1.583, 1.929)	1.298	(1.168, 1.443)	1.284	(1.155, 1.427)
Dyslipidemia	1.145	(1.052, 1.246)	0.908	(0.828, 0.995)	0.895	(0.816, 0.982)
Chronic Liver Disease	1.942	(1.683, 2.241)	1.531	(1.323, 1.771)	1.469	(1.269, 1.702)
Chronic Kidney Disease	4.010	(3.532, 4.553)	2.652	(2.32, 3.032)	2.473	(2.159, 2.833)
Peptic Ulcer Disease	1.511	(1.378, 1.656)	1.153	(1.047, 1.269)	1.105	(1.003, 1.216)
Congestive Heart Failure	2.884	(2.588, 3.215)	1.876	(1.669, 2.109)	1.769	(1.572, 1.991)
Gastroesophageal Reflux	1.217	(1.116, 1.328)	0.951	(0.869, 1.041)	0.819	(0.747, 0.898)
Antiplatelet/Anticoagulant *						
Antiplatelet User	1.217	(1.118, 1.324)	1.062	(0.969, 1.163)	1.025	(0.935, 1.124)
Anticoagulant User	1.501	(1.336, 1.687)	1.274	(1.125, 1.443)	1.218	(1.074, 1.382)
By surgery types						
Head and Neck (major)	7.749	(4.937, 12.163)	7.384	(4.696, 11.609)	4.160	(2.613, 6.622)
Head and Neck (minor)	0.674	(0.402, 1.131)	0.672	(0.401, 1.127)	0.540	(0.321, 0.908)
Cardiovascular (major)	15.011	(11.623, 19.387)	9.218	(7.068, 12.024)	5.760	(4.357, 7.617)
Gastrointestinal (major)	13.629	(10.974, 16.928)	11.093	(8.912, 13.807)	10.010	(7.962, 12.587)
Gastrointestinal (minor)	3.236	(2.522, 4.152)	2.690	(2.094, 3.456)	2.302	(1.785, 2.968)
Genitourinary (major)	3.757	(2.556, 5.523)	2.769	(1.880, 4.079)	2.347	(1.585, 3.476)
Gynecological (major)	0.928	(0.468, 1.840)	0.996	(0.501, 1.980)	1.115	(0.561, 2.219)
Orthopedic (major)	3.497	(2.830, 4.320)	2.906	(2.341, 3.608)	2.300	(1.842, 2.873)
Orthopedic (minor)	1.991	(0.277, 14.332)	1.895	(0.263, 13.669)	1.627	(0.225, 11.783)
Others (minor)	Reference		Reference		Reference	
Postoperative drugs user *						
Acetaminophen/NSAIDs	0.580	(0.533, 0.631)			0.716	(0.653, 0.786)
Cyclooxygenase 2 inhibitor	0.994	(0.881, 1.122)			0.962	(0.840, 1.101)
Anticonvulsant	1.017	(0.854, 1.210)			0.924	(0.771, 1.107)
Antidepressant	0.961	(0.757, 1.221)			0.920	(0.721, 1.174)
Opioid	1.854	(1.698, 2.025)			0.889	(0.807, 0.980)
Steroid	1.641	(1.491, 1.806)			1.421	(1.280, 1.577)
Mucosal Protective Agents	1.844	(1.696, 2.005)			1.902	(1.743, 2.074)
H2 Receptor Antagonist	0.899	(0.825, 0.979)			1.201	(1.097, 1.314)
Proton Pump Inhibitor	3.635	(3.335, 3.962)			2.949	(2.691, 3.233)
Antacid	1.067	(0.980, 1.162)			1.215	(1.108, 1.332)

Model 1 is a nonadjusted analysis for each variable, Model 2 includes baseline characteristics before surgery (age, sex, income, residence, comorbid conditions, and antiplatelet/anticoagulant drug use) and surgery type, and Model 3 additionally included postoperative drug use, including analgesic use and gastrointestinal medication. * Nondrug users and without comorbidities are references.

## Data Availability

The data that support the findings of this study are available from the NHIS. Restrictions apply to the availability of these data, which were used under license for this study. Data are available from the authors with the permission of the NHIS.

## Supplementary Materials

The following are available online at https://www.mdpi.com/article/10.3390/jpm11111222/s1, Table S1: Profiles for the Surgery Categories.
