# Supplementary material for: Risk of Postoperative Gastrointestinal Bleeding and Its Associated Factors: A Nationwide Population-Based Study in Korea
Source: J Pers Med. 2021 Nov 18;11(11):1222. doi: 10.3390/jpm11111222 (PMC8621831; doi:10.3390/jpm11111222)
Supplement: Supplementary file 1 [file jpm-11-01222-s001.zip › jpm-1409441-supplementary.pdf]

Supplementary Table S1. Profiles for the Surgery Categories

Categories	Included Surgeries (examples)
Head and Neck (major)	Skull Base Surgery
	Brain Tumor Surgery
Head and Neck (minor)	Tonsillectomy
	Sinus Surgery (endoscopic/general)
Cardiovascular (major)	Coronary Bypass Surgery
	Cardiac Surgery
	Aneurysmectomy
	Cerebral Aneurysmectomy
Gastrointestinal (major)	Gastrectomy
	Hepatectomy
	Colectomy
	Pancreatectomy
	Pancreatoduodenectomy
	Intestinal Obstruction Surgery
	Proctectomy/Sigmoidectomy
	Small Intestine Resection
	Splenectomy
Gastrointestinal (minor)	Appendectomy
	Cholecystectomy
Genitourinary (major)	Prostatectomy
	Adrenalectomy
	Nephrectomy
	Kidney Transplantation
	Nephroureterectomy
	Cystectomy
Gynecological (major)	Hysterectomy
Gynecological (minor)	Cesarean Section
Orthopedic (major)	Hip Replacement
	Knee Replacement
	General Spinal Surgery
	Artificial Joint Arthroplasty
Orthopedic (minor)	Endoscopic Spinal Surgery
Others (minor)	Varicose Vein Ligation/Surgery
	Inguinal/Femoral Herniorrhaphy
	Partial/Total Mastectomy
	Thyroidectomy
	Hemorrhoidectomy
